# Magnetic Resonance Imaging Used to Define the Optimum Needle Length in Pigs of Different Ages

**DOI:** 10.3390/ani12151936

**Published:** 2022-07-29

**Authors:** Maren Bernau, Ulrike Gerster, Armin Manfred Scholz

**Affiliations:** 1 Livestock Center Oberschleissheim, Veterinary Faculty of the Ludwig-Maximilians-University Munich, St. Hubertusstrasse 12, 85764 Oberschleissheim, Germany; ulrike.gerster@gmx.de (U.G.); armin.scholz@lvg.vetmed.uni-muenchen.de (A.M.S.); 2 Fakultät Agrarwirtschaft, Volkswirtschaft und Management, Hochschule für Wirtschaft und Umwelt Nürtingen-Geislingen, Neckarsteige 6-10, 72622 Nürtingen, Germany

**Keywords:** injection depth, pigs, magnetic resonance imaging, animal welfare

## Abstract

**Simple Summary:**

Injections always cause local reactions. To minimize these, the optimum injection site as well as the optimum injection depth should be considered. A total of 730 magnetic resonance images of pig necks were evaluated to determine the depth from the skin to the presumed optimum injection site in the muscle, and the depth to the spine at two sites at the base of the ear. Further studies are needed to determine the optimum needle length for different age groups and injection volumes.

**Abstract:**

Intramuscular injections result in tissue destruction and alteration. Therefore, it is necessary to evaluate the optimum injection point for intramuscular injections. As animals—especially pigs—vary in size and explicit information about injection depth is not available. To determine the predicted optimum injection depth, magnetic resonance imaging was used in pigs of different ages and weight groups. In total, 730 magnetic resonance images of 136 pigs were used to calculate the optimum injection depth for intramuscular injections. Four age groups were evaluated: <29 days of age, 29–70 days of age, 71–117 days of age and >170 days of age. For fattening pigs (71–117 days of age), the present study recommends a needle length of 20 mm (range: 40–58 mm). For younger pigs (<70 days of age), a needle length of 12 to 14 mm (range: 10–18 mm), and for older pigs (>170 days of age), a needle length of 30 mm (range: 25–37 mm) is recommended. However, more data are needed. Therefore, further studies are necessary, especially in the youngest (suckling pigs) and oldest (sows) age groups, as these are the groups mainly injected/vaccinated. Additionally, age and weight should be examined in more detail compared to fat distribution in the neck, genetics and the sex of the animal.

## 1. Introduction

Each intramuscular injection results in tissue alteration and tissue destruction [1,2,3,4], which very likely results in pain. Tissue alterations based on drug application are described for different formulations and different animal categories [5,6,7]. In contrast to laboratory animals [8,9,10], explicit information on injection type, volume and needle size is missing for farm animals.

Information describing the optimum injection site might lead to less pain and improve efficiency of drug effects in farm animals. Several articles have compared injection types and sites in pigs, i.e., [11,12]. For injecting pigs intramuscularly, the neck tissue behind the base of the ear has been determined to be a suitable injection site [11]. Houpert et al. [11] showed that the muscles in the neck region are smaller and surrounded by loose conjunctive tissue, which reduces the spread of injective material compared with the loin or gluteal region. Song et al. [12] did not find any differences in the intramuscular injection type (neck or thigh area) in piglets with respect to the pharmacokinetic properties. However, repeated injections were not examined in this study [12]. In books for porcine medicine the neck area behind the base of the ear is recommended as the best injection site for intramuscular injections [13,14]. Baumgartner [15] recommended one finger wide, *caudal* from the base of the ear, as the injection site of choice for pigs weighing over 10 kg (for piglets he recommended the neck tissue).

Aside from the injection site, needle length and needle diameter play an important role in both drug delivery and associated pain. It is well known that thinner and shorter needles cause less irritation [16,17]. Human studies have shown a close correlation between injection pain and needle thickness: smaller diameters of needle results in less pain at insertion [17,18,19].

Reiner [14] described the optimum needle length for piglets as 12 to 20 mm, for fattening pigs as 40 mm and sows as 75 mm; a weight range is not described. Houpert et al. [11] concluded that the weight of the animals does not influence the location of the deposit, but they only used a 2 mL volume and therefore qualified their statement, stating that their results might change if larger volumes were used. Additionally, Houpert et al. [11] did not detect an effect of the needle length (30 mm vs. 40 mm). Thomsen et al. [20] found differences in tissue deposition depending on needle length (5 mm vs. 8 mm) using subcutaneous injections in pigs.

The optimum needle length varies depending on the injection site, age and/or weight of the pig, and the injection volume. Therefore, needle length is an important parameter to guarantee the least harmful injection for each animal size, to avoid painful injuries due to wrong drug application such as intraneural or periosteal injections. The aim of this study was to evaluate magnetic resonance images of porcine neck tissue to generate helpful information for predicting the optimum/suitable injection depth for intramuscular injection in pigs of different age and weight groups, injected into the recommended injection site. This study is entirely anatomical in nature and did not involve measurements of needle tip locations nor evaluate parameters that impact the distribution efficiency of injected material in pigs.

## 2. Materials and Methods

### 2.1. Animals and Management

For this study, 730 magnetic resonance images of 136 pigs were used for the calculations. Observations were divided into the four different age groups (see Table 1). Pigs were purebred German Landrace (26% of the animals) or hybrids of Pietrain sires and German Landrace sows (74% of the animals). Male (not castrated; 54% of the animals) and female pigs (46% of the animals) were included (age group >171 days only for female animals).

These images were collected in two experimental studies, both conducted in accordance with the District Government of Upper Bavaria (registration numbers: 55.2-1-54-2532-138-11 and 55.2-1-54-2532-196-2014) and in compliance with local and national guidelines [21,22,23].

### 2.2. Magnetic Resonance Imaging

An open low-field MRI system (Siemens Magnetom Open; 0.2 Tesla magnetic field strength) was used to image the neck region of the pigs. All pigs were anaesthetized for MR scanning to avoid movements and to guarantee an excellent image quality. Anesthesia was performed by a combination of azaperone (2 mg per kg body weight) and ketamine (10–15 mg per kg body weight) given intramuscularly [24] into the hind leg muscles to avoid any tissue changes in the neck region. The neck region of all animals was scanned via MRI using a coronary T1-weighted sequence to maximize contrast between soft tissues. The corresponding sequence parameters are shown in Table 2, divided for the different age groups. All pigs were bedded in a prone position with front limbs flexed and hind limbs extended, to guarantee good bedding for optimal image quality. The procedure was performed up to seven times in a weekly interval for a single animal. In total 730 magnetic resonance images were collected from 136 pigs.

### 2.3. Image Evaluation

**Figure 1:**
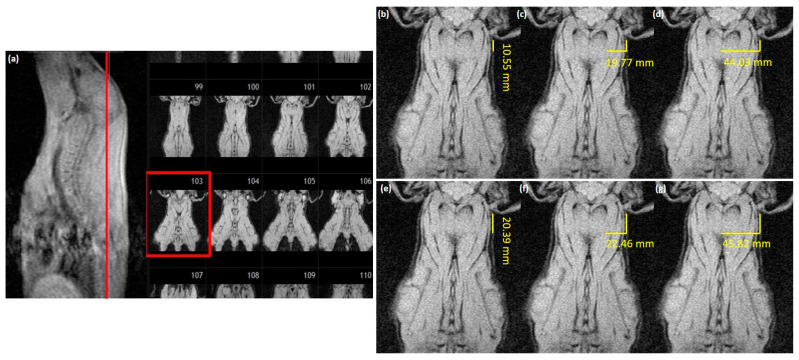
Demonstration of the measurements performed at the ear-base level. (**a**) Definition of the coronary image at the ear-base level (pig 1668; 23.5 kg, 94 days of age). (**b**) Defining the first injection point (OI_1), 10.55 mm behind the base of the ear. (**c**) Measuring the optimum injection point at 10.55 mm distance (OI_1) from the base of the ear (here: 19.77 mm). (**d**) Measuring the distance to the middle of the neck at 10.55 mm distance (MN_1) from the base of the ear (here: 44.03 mm). (**e**) Defining the second injection point (OI_2), 20.39 mm behind the base of the ear. (**f**) Measuring the optimum injection point at 20.39 mm distance (OI_2) from the base of the ear (here: 22.46 mm). (**g**) Measuring the distance to the middle of the neck at 20.39 mm distance (MN_2) from the base of the ear (here: 45.82 mm).

The T1-weighted magnetic resonance images were used to evaluate the presumed optimum injection depth. Synedra View Personal^®^ software (Synedra IT GmbH, AIM 16 “Hermes”) was used to define the coronary magnetic resonance image at the ear-base level (see Figure 1a). After defining the coronary image at the ear-base level, this image was used to measure the optimum injection depth (OI). For intramuscular injection, the *Musculus biventer cervicis* is recommended [25]; therefore, OI was defined as a point in the middle of the *Musculus biventer cervicis*. For distance measurements the Sante Dicom Editor^®^ (Santesoft, Version 3.3.4) was used.

Two “injection sites” were evaluated: first, 10.55 mm behind the base of the ear (see Figure 1b) and, second, 20.39 mm behind the base of the ear (see Figure 1e). Both length measurements (10.55 mm and 20.39 mm distance) were pre-installed lengths in the software. These pre-installed lengths were chosen in order to select the same distance and, thus, to match the recommended injection site one finger wide, *caudal* to the base of the ear [15]. At both injection sites, the optimum injection depth (OI; see Figure 1c,f) and the distance to the middle of the neck (MN; see Figure 1d,g) were measured. MN was measured as the length from the skin to the middle of the neck of a pig (clearly detectable in the magnetic resonance image) in order to evaluate the whole neck depth (including the half of the vertebral body). A total of 730 magnetic resonance images were evaluated and grouped according to the age of the animals.

### 2.4. Statistical Evaluation

Mean distance values and standard deviation values for the four defined age groups (<29 days, 29–70 days, 71–117 days and >170 days) were calculated using Excel software. Additionally, a stepwise regression analysis was performed using the proc reg function of SAS software (version 9.3, SAS Inst. Inc., Cary, NC, USA). Age and weight were included as potential regression variables. The significance level for entry and stay in the model was set at *p* < 0.05.

## 3. Results

The mean values are displayed in Table 3, divided into the four age groups and the four variables: MN_1, OI_1, MN_2, OI_2. The age group < 29 days and the age group 29–70 days received similar results regarding the optimum injection depth (OI) and length to the middle of the neck (MN). For the age group < 29 days (suckling pigs), the OI received a depth of around 12 mm (10–15 mm); for the age group 29–70 days the OI was measured in a depth of around 14 mm (10–18 mm). The length to the middle of the neck (MN) is 36/37 mm (30–40 mm) for both measurement points and for both age groups. For the group 71–117 days of age, the OI received a depth of 20 mm (15–25 mm), and for MN, a length of 45–60 mm was measured. For the oldest group (>170 days), OI was measured in a depth of around 30 mm (25–36 mm) under the skin surface at both measurement points, whilst MN was measured with a length of 75 mm (70–80 mm) (Table 3).

Similar results for the OI and MN were obtained, even if measurements were separated regarding the sexes (see Table S1—Supplementary Materials).

Table 4 demonstrates the results of the stepwise regression analysis including all animals, not separated into age groups. The calculated coefficient of determination was higher for the MN measurement (R^2^ = 0.91–0.92) at both measurement points, as was the case for the OI measurement (R^2^ = 0.71–0.59). Age and weight are included in the regression equations. Additionally, Figure S1 (Supplementary Materials) visualizes these results in a multiple regression.

Table 5 shows the results of the stepwise analysis divided into the four age groups. For the oldest age group (>170 days), at both measurement points, no variable entered the regression equation. Additionally, no significantly contributing variables could be found for the youngest age group (<29 days) regarding OI_2.

It can be stated that, for OI_1, weight had more influence in finding the optimum injection depth when the animals are younger. This can be supported by Table S1. For OI_2, regarding the age group 71–117 days of age, an age effect could be detected.

## 4. Discussion

Some studies exist describing the importance of injection sites and needle length in pigs, i.e., [11,12]. Human and animal research [16,17,18,19,26,27] have shown that the optimum injection depth results in less pain and being transferred to animals might result in higher animal welfare. This is of great importance, even if irritating inoculums or vaccines are used, which can result in large-scale tissue alterations [1,2,5,6,7,25].

1. Optimum needle length: The present study calculated the optimum needle length by measuring the distance from the skin to (a) the defined optimum intramuscular injection point (OI) and to (b) the middle of the neck (MN). The *Musculus biventer cervicis* is recommended for intramuscular injection [25], injecting the needle one finger width *caudal* behind the base of the ear [15]. This can be confirmed by the performed MRI measurements.

Based on the presented results (Table 3), a needle length of 10–18 mm is recommended for piglets < 71 days of age. Therefore, the present study confirms the statement of Reiner [14], with a slightly different range. It has to be considered that most of the measurements (*n* = 307) were taken for age group 29–70 days of age with a mean weight of 13.0 ± 4.1 kg (range 6.0 to 31.5 kg). Therefore, further studies are needed in younger piglets (suckling pigs) to evaluate the optimum needle length and injection volume due to smaller muscles and body sizes. For fattening pigs (71–117 days of age), the present study recommends a needle length of 20 mm (range: 15–25 mm; Table 3). This is in contrast to the recommendation of Reiner [14], who recommended 40 mm. As no age or weight range is given by Reiner [14], there might be some uncertainty. The present measurements showed that the length to the middle of the neck is 45–60 mm (Table 3) in the age group 71–117 days, with a mean weight of 33.0 ± 7.9 kg (weight range from 12.5 to 54.5 kg). Therefore, a needle length of 40 mm might result in a deep intramuscular injection or maybe in a periosteal or intraneural injection, which results in painful tissue alteration or functional nerve injury [28]. The age group of > 170 days resulted in an optimum needle length of 30 mm (range: 25–37 mm), with a 70–80 mm length to the middle of the neck. This group had a mean weight of 87.9 ± 11.7 kg, which might be similar to the fattening group in the publication of Reiner [14]. Adult pigs, sows or boars, were not included in this study. This is due to the size of the tube or the diameter of the gantry using the three-dimensional imaging tool available, which limits the size of the animal that can be examined [29].

It has to be taken into account that length to the middle of the neck (MN) does not represent the length from the skin to a vertebral body. Therefore, further studies are needed, to detect the length from the skin at the optimum injection site to the vertebral body, in order to determine the best needle length that avoids painful periosteal or intraneural injections [28]. As shown in this study, MRI seems to be an appropriate tool to calculate the optimum injection depth and, with this, needle length, in pigs with respect to their body size, as it is able to visualize muscle tissue and body contours in a non-invasive way [29,30,31,32,33,34,35,36,37]. 

2. Weight as a factor for optimum needle length: The fact that weight plays an important role for optimum injection [1,11] can be inferred from the present results, as shown in the stepwise regression (Table 5) where weight is always (except for OI_2) an independent variable, with a higher impact in younger animals. As weight plays an important role, further studies are needed to evaluate the impact on needle length in more detail. Beside this, sex and sex-specific growth patterns, and fat distribution patterns should be studied, as well as genetic effects. Sex influences total body fat and subcutaneous adipose tissue [32,36,37], which might influence the optimum injection point and the optimum needle length. The present study did not find differences in sex or genetics (Table S1), but it only examined 136 animals in four age groups that were mainly Pietrain hybrids (74%).

Additionally, the volume of injectable solutions may influence the location and the local reaction [1,2,5,25]. Therefore, muscle growth should be taken into account as well. In pigs > 29 days of age, OI_2 is smaller than OI_1 (Table 3). This might be explained by anatomic orientation of the muscle and muscle growth, as this seems to be different in pigs under 29 days of age. Due to the small number of animals < 29 days of age used in this study compared to the other groups, this should not be overestimated.

3. Measurement method: Measurement point 1 (_1; Figure 1b) was defined as 10.55 mm behind the ear base and measurement point 2 (_2) was defined as 20.39 mm behind the ear. As the optimum injection point in pigs is defined as “behind the base of the ear” [13,14] or “one finger wide *caudal* behind the base of the ear” [15], the Sante Software fixed measurement length was used to ensure the same distance to the ear base for all measured images. Additionally, the measured points should be useful *in praxis* as well. With the chosen fixed measurements, this transfer is around one finger width (_1) or two finger widths (or one rather wide finger (_2)) behind the base of the ear.

The MRI method worked well for the chosen age groups and the chosen MRI sequences were a good compromise between the time of acquisition and image resolution. Therefore, the method and sequence can be recommended for further examinations. Additionally, the positioning of the animal can be recommended for further studies in this context because lying in prone position does not compress neck tissue and flexed front limbs stretch the neck; this is more natural than having extended front limbs, which might compress the lower neck tissue.

4. Further studies needed: Using three-dimensional imaging devices, such as MRI, can help to predict the optimum needle length and, furthermore, help to evaluate the optimum needle thickness. This study confirms the possibility of three-dimensional imaging and the usefulness of analyzing images in different research activities. For upcoming questions, MRI can be used as a method receiving close relationships with pathomorphological data [1,2,38]. Muscle growth can also be detected using MRI [29,32,36] and should be analyzed in further studies with respect to sex, weight and genetics.

## 5. Conclusions

The aim of this study was to use magnetic resonance images to determine the optimum needle length for use in pigs of different age groups, to give recommendations for practitioners and, with this, to help achieve effective but minimally painful injections. The results showed that MRI can be used to calculate the optimum injection depth (= optimum needle length) in various age groups. Compared with previous studies, the present results confirm an optimum needle length of 10–18 mm (Reiner [14]: 12–20 mm) for piglets <70 days of age. For older pigs, a 20 mm needle length (age group: 71–117 days of age) and a 30 mm needle length (age group >170 days of age) were calculated. However, further studies in defined age and weight groups are necessary, especially in the youngest (<29 days of age; suckling pigs) and the oldest (>170 days of age; sows) age groups, as these are the groups mainly vaccinated/injected on farms. Additionally, the impact of injection volume and needle size should be evaluated as well. Finally, the injection site should be standardized for measurements. Measurements post-injection can help identify the optimum injection depth in various animal sizes and injection volumes.

## Figures and Tables

**Table 1:** Information regarding the different age and weight groups in detail.

Age Group [d]	Number of Observations	Mean Age [d]	Age Range from–to [d]	Mean Weight [kg]	Weight Range from–to [kg]
<29	62	21.6 ± 5.9	5–26	7.0 ± 2.0	3.5–11.5
29–70	307	50.3 ± 11.1	31–70	13.0 ± 4.1	6.0–31.5
71–117	219	94.2 ± 12.6	71–117	33.0 ± 7.9	12.5–54.5
>170	142	179.3 ± 7.1	171–195	87.9 ± 11.7	58–124

**Table 2:** MRI imaging parameter.

MRI Parameter	Age Group		
	Suckling Pigs <29 Days of Age	Weaned Pigs 29–70 Days of Age	Fattening Pigs >71 Days of Age
TR [msec]	814	814	814
TE [msec]	17	17	17
Pixel size	1.30 × 0.70	1.67 × 0.90	2.54 × 1.37
FOV [mm]	180	230	350
Matrix	138 × 256 (54%)	138 × 256 (54%)	138 × 256 (54%)
Number of slices	22	22	22
Slice thickness [mm]	4	4	5
Distance factor	0.5	0.5	1.00
Examination time	5 min 40 s	5 min 40 s	5 min 40 s

**Table 3:** Mean values including standard deviations for the optimum injection point (OI) and the length to the middle of the neck (MN) at first (_1) and second (_2) measurement point for each of the four defined age groups.

Age Group [d]	n	MN_1 [mm] Mean ± SD	OI_1 [mm] Mean ± SD	MN_2 [mm] Mean ± SD	OI_2 [mm] Mean ± SD
**<29**	62	36.3 ± 4.5	12.5 ± 3.4	37.7 ± 4.5	13.7 ± 2.7
**29–70**	307	36.9 ± 4.7	14.7 ± 3.6	37.7 ± 4.4	14.2 ± 3.2
**71–117**	219	51.9 ± 6.5	22.2 ± 4.4	53.7 ± 6.7	20.9 ± 5.2
**>170**	142	74.3 ± 5.3	30.9 ± 5.4	77.6 ± 4.9	29.2 ± 7.3

**Table 4:** Results of the stepwise regression analysis for all observations.

Dependent Variable	Intercept	Slope	Independent Variable	R^2^	RMSE [mm]
OI_1	10.0089	+0.0932 +0.0569	age [d] weight [kg]	0.71	4.14
MN_1	29.3863	+0.1014 +0.3173	age [d] weight [kg]	0.91	4.58
OI_2	10.3903	+0.0774 +0.0611	age [d] weight [kg]	0.59	4.82
MN_2	30.0477	+0.1007 +0.3483	age [d] weight [kg]	0.92	4.57

**Table 5:** Results of the stepwise regression analysis divided into the four age groups and separated to measurement points 1 and 2.

Dependent Variable	Age Group [d]	n	Intercept	Slope	Independent Variable	R^2^	RMSE [mm]
OI_1	<29	62	7.23607	+0.75155	weight [kg]	0.20	3.05
OI_1	29–70	307	9.24606	+0.41847	weight [kg]	0.23	3.21
OI_1	71–117	219	13.93949	+0.25050	weight [kg]	0.20	3.96
OI_1	>170	142	-				
OI_2	<29	62	-				
OI_2	29–70	307	12.04712	+0.16433	weight [kg]	0.05	3.13
OI_2	71–117	219	6.94691	+0.14840	age [d]	0.13	4.82
OI_2	>170	142	-				

## Data Availability

Not applicable.

## Supplementary Materials

The following supporting information can be downloaded at: https://www.mdpi.com/article/10.3390/ani12151936/s1, Figure S1: Multiple regressions showing the accuracy of using age [d] and weight [kg] to estimate the depth of the optimum injection site 10.55 mm (OI_1; Figure S1a) and 20.39 mm (OI_2; Figure S1b) behind the base of the ear. Multiple regression showing the accuracy of using age [d] and weight [kg] to estimate the depth to the spine 10.55 mm (spine_1; Figure S1c) and 20.39 mm (spine_2; Figure S1d) behind the base of the ear. Table S1: Mean values including standard deviations for the optimum injection point (OI) and the length to the middle of the neck (MN) at first (_1) and second (_2) measurement point, divided into the four defined age groups and separated to sex with mean age and weight.
