# Supplementary material for: Magnetic Resonance Imaging Used to Define the Optimum Needle Length in Pigs of Different Ages
Source: Animals (Basel). 2022 Jul 29;12(15):1936. doi: 10.3390/ani12151936 (PMC9367419; doi:10.3390/ani12151936)

Supplementary material:

Table 3S: Mean values including standard deviations for the optimum injection point (OI) and the length to the middle of the neck (MN) at first (_1) and second (_2) measurement point, divided into the four defined age groups and separated to sex with mean age and weight.

Age group [d]	sex	n	Mean age [d]	Mean weight [kg]	MN_1 [mm] Mean ± SD	OI_1 [mm] Mean ± SD	MN_2 [mm] Mean ± SD	OI_2 [mm] Mean ± SD
**< 29** (n=62)	male	29	22.7 ± 5.2	7.2 ± 1.9	35.7 ± 4.6	12.4 ± 2.7	38.4 ± 4.6	13.8 ± 3.2
	female	33	20.7 ± 6.4	6.8 ± 2.1	35.7 ± 4.5	12.6 ± 4.0	37.0 ± 4.4	13.6 ± 2.3
**29-70** (n=307)	male	158	50.1 ± 11.0	12.8 ± 4.0	36.7 ± 4.6	14.5 ± 3.5	37.6 ± 4.3	14.3 ± 3.3
	female	149	50.6 ± 11.2	13.3 ± 4.2	37.1 ± 4.7	15.0 ± 3.7	37.8 ± 4.6	14.1 ± 3.1
**71-117** (n=219)	male	203	95.6 ± 11.8	33.9 ± 7.3	52.6 ± 6.1	22.3 ± 4.3	54.4 ± 6.2	21.1 ± 5.1
	female	14	74.1 ± 2.5	20.7 ± 4.1	42.2 ± 3.3	20.3 ± 5.9	42.6 ± 3.8	19.0 ±5.1
**> 170** (n=142)	male	0						
	female	142	179.3 ± 7.0	87.9 ± 11.7	74.3 ± 5.3	30.9 ± 7.0	77.6 ± 4.9	29.2 ± 7.3

Supplementary material:

Figure S1: Multiple regressions showing the accuracy of using age [d] and weight [kg] to estimate the depth of the optimum injection site 10.55 mm (OI_1; Fig. 2a) and 20.39 mm (OI_2; Fig. 2b) behind the base of the ear. Multiple regression showing the accuracy of using age [d] and weight [kg] to estimate the depth to the spine 10.55 mm (spine_1; Fig. 2c) and 20.39 mm (spine_2; Fig. 2d) behind the base of the ear.

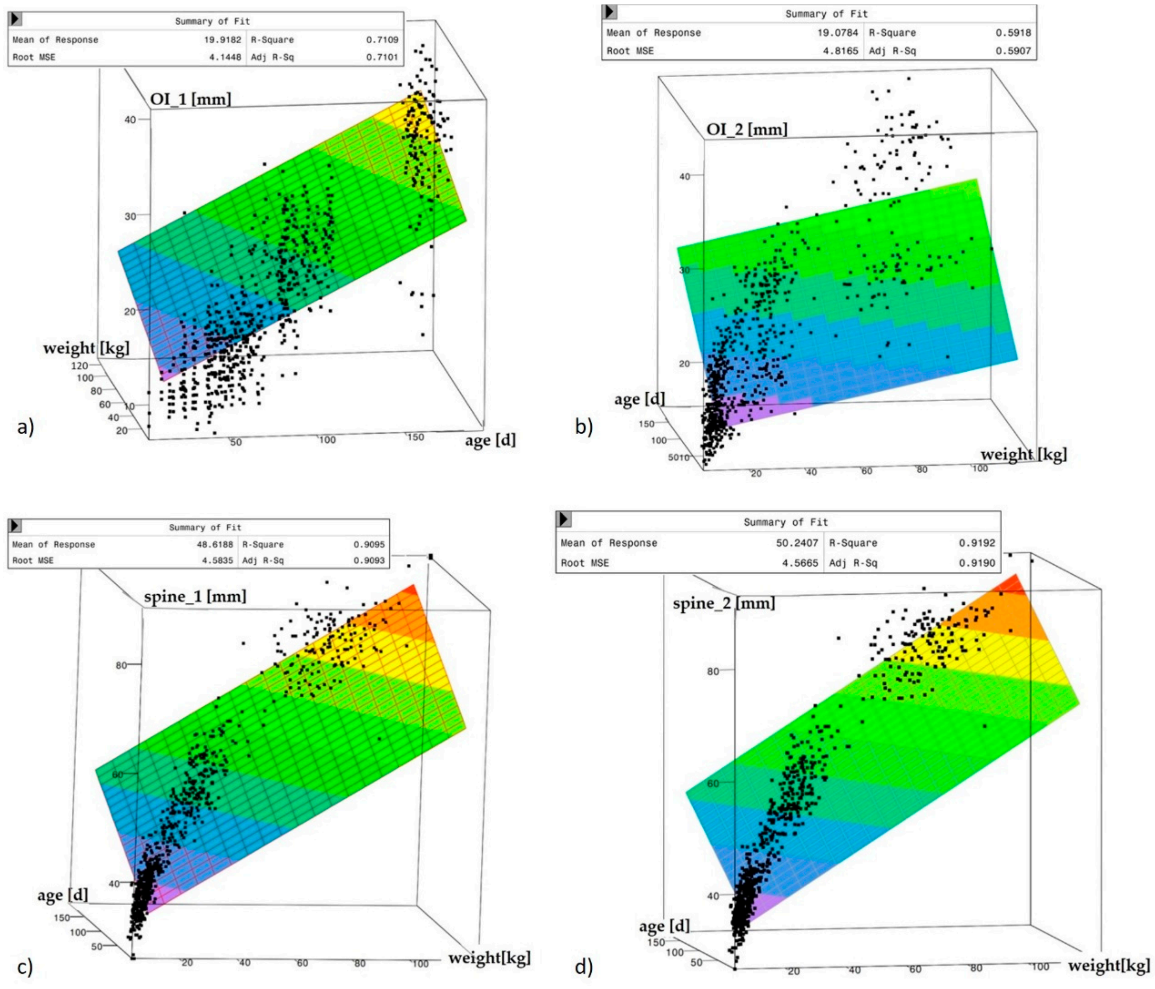

Supplement: Supplementary file 1 [file animals-12-01936-s001.zip › animals-1816140-supplementary.pdf]
